# A European artificial intelligence sovereignty agenda for healthcare

**DOI:** 10.2340/17453674.2026.46809

**Published:** 2026-09-09

**Authors:** Li FELLÄNDER-TSAI, Amy LOUTFI, Peter LANDELL, Anna FELLÄNDER, Fredrik HEINTZ

**Affiliations:** 1 Department of Trauma, Emergency Surgery and Orthopedics, Karolinska University Hospital, Stockholm; 2 Division of Orthopedics and Biotechnology, Department of Clinical Science, Intervention and Technology, Karolinska Institutet, Stockholm; 3 School of Science and Technology, Örebro University, Örebro; 4 AI, Robotics and Cybersecurity Center (ARC), Örebro University, Örebro; 5 Research Institute of Sustainable AI, Stockholm; 6 Örebro University, Örebro; 7 Department of Computer and Information Science (IDA), Linköping University, Linköping; 8 Artificial Intelligence and Integrated Computer Systems (AIICS), Linköping University, Linköping, Sweden

The purpose of this perspective is twofold: first, to highlight artificial intelligence (AI) implementation and patient safety, and second, a broader policy perspective on European AI sovereignty. European AI sovereignty and the access to trustworthy AI systems are highly relevant for orthopedic applications including clinical infrastructure, data governance, medical device regulation, and implementation in clinical workflows. AI has enabled disruptive technological advancements in medicine. AI-assisted navigation, robotics, and image-based diagnostics facilitate the next level of precision-based medicine. Medical documentation and coding based on AI have created hopes of reduced workload and improved working conditions in clinical production.

Fracture detection using AI has demonstrated diagnostic benefits among less experienced human image readers in high-volume emergency settings [1]. AI-based computer tomography-radiostereometric analysis (CT-RSA) has demonstrated accuracy comparable to model-based RSA, apart from providing accurate and predictable preoperative implant planning [2]. Large language models (LLMs) have been shown to generate orthopedic discharge documents faster than humans, maintaining comparable quality [3], and perform on par with or above junior residents in orthopedic exams, highlighting its potential as a learning and educational tool [4].

## Human factors and AI—a devastating case report

Patient safety can be challenged when AI becomes a third party to account for. This can be illustrated by the following real-world case.

*An elderly patient contacts the primary care facility due to a fall with subsequent pain and reduced mobility in the left shoulder. The patient is referred for an emergency radiological examination of the left shoulder. An AI algorithm identifies an undisplaced proximal humerus fracture. The radiologist examines the radiographs and confirms the diagnosis. No suspicion of a dislocation is mentioned in the radiology report. The patient is referred to the emergency department where an emergency care physician who admits lack of experience in interpreting radiographs trusted the radiology report. The attending orthopedic surgeon is consulted and after being informed that the radiographs show an undisplaced proximal humerus fracture recommends follow-up after 2 weeks. At this point, plain radiographs reveal an anterior glenohumeral dislocation. Reassessment of the initial radiographs confirms the diagnosis as already present when the patient presented 2 weeks earlier. The patient developed a full-thickness rupture of the rotator cuff with severely decreased range of motion.*

There are several lessons to be learned from this case of avoidable patient harm involving both active failures and latent conditions. It is obvious that AI should be a support, not a replacement in diagnostic procedures. The case clearly demonstrates the risk of anchoring bias that AI may cause, i.e., that the initial findings affect further assessments, with devastating consequences for the patient. The case also raises questions as to whether radiographic assessments will be less meticulous when AI has already pointed out a finding, leading to a decreased propensity to challenge the initial finding and to assess the whole picture. It also demonstrates that tele-counselling without access to the radiological images may lead to an increased risk of inaccurate assessment and therefore represent a serious patient safety risk.

Clinical assessment of radiographs is an important competence in emergency care, irrespective of AI support tools, especially in musculoskeletal injuries where initial triage in the emergency department does not capture the downstream crippling consequences. Increased awareness of these challenges and the risk of anchoring bias when introducing AI as a support tool is needed. It is urgent to introduce a new chapter in medical education and training including European validation thresholds as well as clearing out of blurred liabilities and legal consequences.

## Technology push, accountability, and regulation

Apart from being a game changer regarding human factors in medicine, AI relies heavily on technology push on a global scale. Technology push means that the technology applied is considered as the most promising solution to an existing issue. The EU likes to talk about “strategic autonomy.” Yet, with AI, there is a risk of quietly sleepwalking into digital dependency. A handful of technology giants currently control the large language models that increasingly mediate what we read, see, and believe. When a few actors with variable accountability own the infrastructure of meaning, this is not just a sign of market failure—it may represent a democratic crisis. The vast majority of graphics processing unit (GPU) computer power is currently non-EU-owned.

News media have long carried legal and moral obligations to serve the public interest. In 2026, the Pope issued a warning that AI is a new Tower of Babel [5], a statement rocking the epicenter of AI with rapid dismissals [6], moving the ethical and moral discussions to new heights, including the question of artificial general intelligence. Generative AI can produce and curate content on a far greater scale, without necessarily having any equivalent duty of care. We cannot reliably distinguish between human and AI-generated material, and we know that today’s foundation models may amplify bias, trample intellectual property rights (IP) and privacy, manipulate human behaviors, and optimize solely for engagement and efficiency.

Beyond these risks, the growing autonomy of AI agentic systems is beginning to reshape markets and institutions themselves. Non-human agents are not only executing decisions but increasingly influencing how value is created, how resources are allocated, and how economic activity is coordinated. In this sense, they are becoming powerful actors that shape the future of our society. Without proper control, this could lead to systems focused more on machine efficiency than on human needs and innovation.

The result is a knowledge infrastructure that is structurally misaligned with European law, values, and democratic norms.

Brussels has already chosen an answer: regulate. The EU AI Act [7], together with the Medical Device Regulation (MDR) and the General Data Protection Regulation (GDPR), has a significant impact on day-to-day-clinical practice in Europe, sets important horizontal rules, and creates a new supervisory architecture for powerful models. But regulation without capability is a recipe for dependence. Europe will not negotiate from a position of strength if the critical models, data pipelines, and chips are all designed and controlled elsewhere.

Without sovereign AI capacity, Europe risks becoming a “rule-taker” that writes guidelines for systems it neither builds nor truly understands.

A sharper and more realistic path than trying to clone the biggest frontier models is emerging: open-source, high-performance models that Europe can actually own, govern, and export. There is a huge underserved market for models that are:

- small enough to run on local or edge infrastructure;
- open and inspectable;
- tailored to specific languages, sectors, and public-interest tasks.

These models are cheaper, faster, more energy efficient, and better suited to Europe’s decentralized economy of small and medium enterprises (SMEs), hospitals, universities, schools, and public administrations than hyperscale black boxes. They can also be designed to embed democratic values as a training objective.

Initiatives such as emerging multilingual open models and scattered research consortia show that it is technically feasible to train competitive small models on high-quality, domain-specific datasets. EuroLLM [8] provides an example of how open, multilingual language models can be developed through a distributed European research consortium, demonstrating the feasibility of building competitive AI systems based on high-quality and linguistically diverse datasets. This can unlock innovation in robotics, process automation, and consumer-facing services including healthcare, while enabling on-premise deployments that actually respect data protection and security requirements. Even if there is a vision, the problem is both fragmentation and underfunding.

## An AI sovereignty agenda for Europe

The rapid pace of AI development means that a few superpowers and middle powers have the scale and capabilities to sustainable sovereignty over time. So what does an AI sovereign agenda for Europe need? First, a central, EU-level funding vehicle dedicated to open small models, with long-term mandates rather than short project cycles. Second, a shared computer, data, and algorithmic infrastructure that universities, SMEs, and public bodies can use without begging for API access from foreign platforms. The AI Factories is a promising start, but more is needed to achieve this.

Third, governance-by-design: practical tooling for AI Act compliance, risk management, and auditability built directly into the model lifecycle, not added as a legal fig leaf at the end.

AI has great societal potential, including both research and academic reporting. As humans, we are free to decide and embrace the vision of the potentials that AI offers. We need a coalition of like-minded democracies around open, human-centered AI—a counterweight to both surveillance-authoritarian and hyper-extractive AI models. For Europe, we either need to accept the role of being a digital consumer economy or invest in our own AI capabilities—starting with small, open models that both protect the public interest and catalyze innovation on European terms. This would also benefit the healthcare sector. The choice seems simple. It is time for Europe to turn away from strategic denial.

## Notes

Disclosures – complete disclosure of interest forms according to ICMJE are available on the article page, doi: 10.2340/17453674.2026.46809

Handling co-editor: Bart Pijls

*Acta* thanks Jacobien Oosterhoff and Aleksi Reito for help with peer review of this manuscript.
